# Transient global ventricular dysfunction in an adolescent affected by pancreatic adenocarcinoma

**DOI:** 10.1186/s12887-016-0628-4

**Published:** 2016-07-19

**Authors:** Maria Debora De Pasquale, Angela Mastronuzzi, Luigi De Sio, Annalisa Serra, Chiara Grimaldi, Marcello Chinali, Ugo Giordano

**Affiliations:** Department of Pediatric Hematology/Oncology and Stem Cell Transplantation, Bambino Gesù Children’s Hospital, Piazza Sant’Onofrio, 4, 00165 Rome, Italy; Pediatric Surgery and Transplantation, Rome, Italy; Pediatric Cardiology, Bambino Gesù Children’s Hospital, Rome, Italy

**Keywords:** Takotsubo cardiomyopathy, Pediatrics, Pancreatic carcinoma, Reversible left ventricular dysfunction

## Abstract

**Background:**

Takotsubo cardiomyopathy (TC) is characterized by a transient decrease in ejection fraction and a reversible left ventricular dysfunction. The pathophysiology of TC is not completely understood. Heterogeneous and multifactorial mechanisms are involved: drugs, emotional and physical stress, genetic and hormonal factors.

**Case presentation:**

A 17 year-old male with metastatic pancreatic adenocarcinoma, under chemotherapy containing 5-fluorouracil, presented severe left ventricular dysfunction requiring mechanical ventilation and inotropes administration. He completely recovered in 2 weeks.

**Conclusion:**

To our knowledge this is the first report of transient form of ventricular dysfunction, mimicking TC, in an adolescent. We believe that children and adolescents receiving 5-fluorouracil should be closely monitored and referred for investigation if they develop cardiac symptoms.

**Electronic supplementary material:**

The online version of this article (doi:10.1186/s12887-016-0628-4) contains supplementary material, which is available to authorized users.

## Background

Takotsubo cardiomyopathy is a reversible cardiomyopathy characterized by a transient decrease in ejection fraction and a reversible left ventricular dysfunction. The pathophysiology of TC is not completely understood. Heterogeneous and multifactorial mechanisms are involved: drugs, emotional and physical stress, genetic and hormonal factors. TC is most often seen in elderly women and has never been reported in an adolescent [1].

## Case Presentation

A 17 year-old male was diagnosed with metastatic pancreatic adenocarcinoma and started on chemotherapy with Oxaliplatin and Gemcitabine. He received the first two courses without any complications. The chemotherapy schedule was then modified to include FOLFIRINOX, on the basis of published data regarding the superiority of this drug combination compared to gemcitabine for the treatment of advanced pancreatic carcinoma [2]. He then received oxaliplatin, 85 mg/m^2^, followed by leucovorin, 400 mg/ m^2^, irinotecan, 180 mg/ m^2^ and 5-fluorouracil (5-FU) at a dose of 400 mg/m^2^ by intravenous bolus, followed by a continuous intravenous infusion of 2400 mg/m^2^ over a 46-hour period. Approximately 4 h after the start of the first 5-FU infusion, the patient experienced vomiting and chest pain as well as an altered level of consciousness with extreme agitation. Progressive diuresis contraction was also recorded. Vital signs were: blood pressure 112/87 mmHg, heart rate 134/min and regular. Brain and thorax CT scans and abdominal x-ray were normal. Monitoring of vital signs revealed a progressive decrease of blood pressure down to 90/50 mmHg. Echocardiography showed severe and diffuse LV hypokinesis with an ejection fraction of 20 %. Moderate right ventricular hypokinesis and a 20 × 16 mm thrombus in the right atrium were also revealed (Additional file 1: Clip 1). Plasma level of B-type natriuretic peptide was 800 pg/ml (normal value 0–100), while creatine kinase-MB, myoglobin and troponin levels were normal. Seventy-two hours later troponin and myoglobin levels rose to 0.16 ng/ml and 356 ng/ml respectively (normal values 0-0.10 and 0–170 respectively).

The patient was then transferred to the intensive care unit and placed on mechanical ventilation. Treatment with milrinone 0.75 mcg/kg/min, dopamine 12 mcg/kg/min, and epinephrine 0.05 mcg/kg/min was started together with anticoagulation based on heparin 20 UI/kg/h. Twenty-four hours later echocardiography showed no improvement in LV systolic and diastolic function. Levosimendan 0.1 mcg/kg/min and norepinephrine 0.06 mcg/kg/min were added while dosage of milrinone was decreased to 0.5 mcg/kg/min. Dobutamine and fenoldopam were also used. Vital signs improved slowly and echocardiography showed a progressive improvement in ventricular systolic and diastolic function (Additional file 2: Clip 2) with complete recovery after 14 days (Additional file 3: Clip 3). Under anticoagulation with low molecular weight heparin, the right atrial thrombus disappeared after 3 months. The final diagnosis was: reversible cardiomyopathy induced by 5-FU. Consequently, the chemotherapy schedule was modified and 5-FU administration excluded.

## Discussion

We report a case of reversible cardiomyopathy induced by 5-FU. Takotsubo cardiomyopathy (TC), also known as apical ballooning syndrome, is a quite new clinical entity characterized by a transient decrease in ejection fraction and a reversible LV dysfunction consisting of dyskinesia involving the apical or midventricular segments. TC is most often seen in elderly women. In a review of studies from 2000 to 2012, Bossone et al reported that the age of patients affected by TC ranged from 59 to 73 years [1]. The pathophysiology of TC has not been fully identified. Heterogeneous and multifactorial mechanisms are involved [3–7]. Emotional and physical stress may play a role by releasing circulating catecholamines that have direct effects on the myocardium. The mechanism underlying the association between sympathetic stimulation and myocardial stunning is unknown. One possibility is ischemia resulting from epicardial coronary arterial spasm. An alternative mechanism is microvascular spasm. Abnormal coronary flow in the absence of obstructive disease has been reported in patients with stress-related myocardial dysfunction. A third possible mechanism of catecholamine-mediated myocardial stunning is direct myocyte injury, through cyclic AMP–mediated calcium overload or by producing oxygen-derived free radicals [4]. Familial cases of TC are also described, suggesting a genetic role in the pathogenesis of this condition [5]. However, estrogen deficiency may also be a risk factor in the development of this type of reversible cardiomyopathy. This deficiency could explain the high incidence in post-menopausal women [6].

Several reports have discussed drugs that may cause TC [3, 7]. Among these, 5-FU is described as a potential cause. Cardiotoxicity is a well-known adverse effect of 5-FU which occurs in 1.2 to 18 % of patients, but the mechanism involved is still not fully understood [8, 9]. In recent years several cases of 5-FU-induced Takotsubo-like syndrome have been reported in the literature [10–12]. It has been hypothesized that extreme sympathetic stimulation causing coronary vasospasm may play a role in the development of TC.

According to the reported cases, TC is often seen in elderly women who frequently have histories of smoking, alcohol abuse, anxiety states, and hyperlipidemia. Our patient however, was an adolescent, with no previous history of cardiac dysfunction or other reported risk factors. He was under treatment for pancreatic adenocarcinoma with liver metastases and, 4 h after the start of 5-FU infusion, he developed chest pain and an altered level of consciousness, which are typical findings associated with TC. Echocardiography showed severe and diffuse LV hypokinesis with an increased level of cardiac enzyme B-Type natriuretic peptide, troponin and myoglobin, leading us to hypothesize TC. To our knowledge this is the first reported case of 5-FU- induced transient form of ventricular dysfunction, mimicking TC, in an adolescent. This is probably due to the fact that the drugs recognized as possible triggers of TC are not currently used in pediatric and young populations affected by cancer [3].

The chemotherapeutic agent 5-FU, by inhibiting thymidylate synthase in malignant cells, disrupts DNA synthesis and promotes cell death. It is currently used as adjuvant chemotherapy for colorectal cancer and frequently for the treatment of pancreatic, breast, bladder, gastric, esophageal and prostate cancer [8]. These neoplasms rarely affect adolescents. In the USA, the annual age-adjusted incidence rate for all carcinomas in patients younger than 20 years is 1.4 per million. More specific figures for pancreatic cancers in this age group are unavailable [13]. The only occasional use of 5-FU in the pediatric population could explain the absence of reports of TC in this age group. This is in fact the first observation of Takotsubo-like syndrome, in an adolescent.

## Conclusion

Although TC has only been reported in adults, children and adolescents with cardiac symptoms during chemotherapy with 5-fluorouracil should be closely monitored and referred for further investigations if there is suspicion of this condition. Prompt acute clinical assessment coupled with ECG recordings and cardiac enzyme analysis should be performed if cardiotoxicity is suspected. Further studies are necessary to be able to assess the real incidence of TC in the pediatric population.

## Abbreviations

5-FU, 5-fluoruracil; LV, left ventricular; TC, takotsubo cardiomyopathy

## Additional files

Additional file 1: Clip 1. Exam performed on May 17th. Diffuse left ventricular hypokinesia can be observed with apical dyskinesia and dilation. (AVI 17411 kb) Additional file 2: Clip 2. Exam performed on May 23^rd^. Improvement in left ventricular function is observed, with mild diffuse hypokinesia and normal left ventricular geometry. (AVI 6992 kb) Additional file 3: Clip 3. Exam performed after 6 months. Normal left ventricular geometry and function is restored. (MPEG 1612 kb)
